# Marked reduction in antibiotic usage following intensive malaria control in a cohort of Ugandan children

**DOI:** 10.1186/s12916-021-02167-2

**Published:** 2021-11-30

**Authors:** Paul J. Krezanoski, Michelle E. Roh, John Rek, Joaniter I. Nankabirwa, Emmanuel Arinaitwe, Sarah G. Staedke, Susan Nayiga, Michelle S. Hsiang, David Smith, Moses Kamya, Grant Dorsey

**Affiliations:** 1 University of California, 1001 Potrero Avenue, San Francisco, CA 94118 USA; 2 Infectious Diseases Research Collaboration, Kampala, Uganda; 3 Makerere University College of Health Sciences, Kampala, Uganda; 4 London School of Hygiene and Tropical Medicine, London, UK; 5 University of Washington, Seattle, WA USA

**Keywords:** Malaria control, Pediatric infectious diseases, Antibiotic prescriptions, Malaria, Antibiotic usage, Prevention, Pediatrics

## Abstract

**Background:**

Intensive malaria control may have additional benefits beyond reducing the incidence of symptomatic malaria. We compared antibiotic treatment of children before and after the implementation of highly effective malaria control interventions in Tororo, a historically high transmission area of Uganda.

**Methods:**

Two successive cohorts of children, aged 0.5 to 10 years, were followed from September 2011 to October 2019 in a dedicated study clinic. Universal distribution of long-lasting insecticidal nets was conducted in 2013 and 2017. Sustained indoor residual spraying of insecticide (IRS) was initiated in December 2014. Generalized linear mixed-effects models were used to compare the incidence of antimalarial and antibiotic treatments before and after vector control measures were implemented.

**Results:**

Comparing the period prior to the implementation of IRS to the period after IRS had been sustained for 4–5 years, the adjusted incidence of malaria treatments decreased from 2.68 to 0.05 per person-year (incidence rate ratio [IRR] = 0.02, 95% CI 0.01–0.03, *p* < 0.001), and the adjusted incidence of antibiotic treatments decreased from 4.14 to 1.26 per person-year (IRR = 0.30, 95% CI 0.27–0.34, *p* < 0.001). The reduction in antibiotic usage was primarily associated with fewer episodes of symptomatic malaria and fewer episodes of fever with sub-microscopic parasitemia, both of which were frequently treated with antibiotics.

**Conclusions:**

In a historically high transmission setting, the implementation of highly effective vector control interventions was followed by a marked reduction in antibiotic treatment of children. This added benefit of malaria control could have important implications for antibiotic prescribing practices, efforts to curtail antimicrobial resistance, and health system costs.

**Supplementary Information:**

The online version contains supplementary material available at 10.1186/s12916-021-02167-2.

## Background

Intensive malaria control, including widespread distribution of long-lasting insecticidal nets (LLINs) and indoor residual spraying (IRS) of insecticides, can significantly lower the burden of malaria in endemic settings [1]. Vector control interventions aimed at reducing transmission intensity can reduce malaria burden, but reducing malaria may have additional health benefits beyond those attributable to malaria alone [2, 3]. We hypothesized that intensive vector control in a previously high-transmission setting, resulting in the near elimination of malaria, would be associated with reduced antibiotic use among children.

Acute bacterial and viral infections are common in malaria-endemic settings, and malaria may affect the risk of co-infection with these other pathogens [4, 5]. Moreover, decisions about the use of antibiotics during clinical encounters are inextricably entwined with the burden of malaria in a community [6]. This interrelationship is most apparent during febrile clinical encounters, but even infection with malaria parasites in the absence of fever (i.e., asymptomatic parasitemia) may have an effect on the likelihood of clinical presentation resulting in the use of antibiotics. The widespread use of antibiotics to treat non-malarial infections and the concomitant use of antibiotics in patients treated with antimalarial drugs may be unnecessary and may contribute to the selection of antimicrobial resistance, which is a major public health threat [7–9]. The effect of malaria control on malaria-specific outcomes and the interaction between malaria diagnoses and antibiotic use have been well-researched, but evidence on the use of antibiotics in the context of successful population-level malaria control is limited.

This observational study explores the temporal relationship between the implementation of highly effective malaria control interventions and antibiotic usage in two sequential cohorts of children aged 0.5 through 10 years in Tororo, Uganda. Potential mechanisms for observed trends are explored by stratifying analyses according to the history of fever and the presence or absence of malaria parasites on the day antibiotics were prescribed and summarizing categories of non-malaria diagnoses and antibiotics used.

## Methods

### Study setting

The study took place in Nagongera sub-county, Tororo district, Uganda, historically a high transmission setting currently under intensive vector control, as previously described [1]. Universal distribution of free LLINs occurred in November 2013 and May 2017. IRS with the carbamate bendiocarb began in the entire Tororo district in December 2014, with additional rounds in June and November 2015. In June 2016, the IRS formulation was changed to organophosphate pirimiphos-methyl (Actellic), with repeated rounds in June 2017, June 2018, and March 2019.

### Cohort enrollment and follow-up procedures

**Fig. 1:**
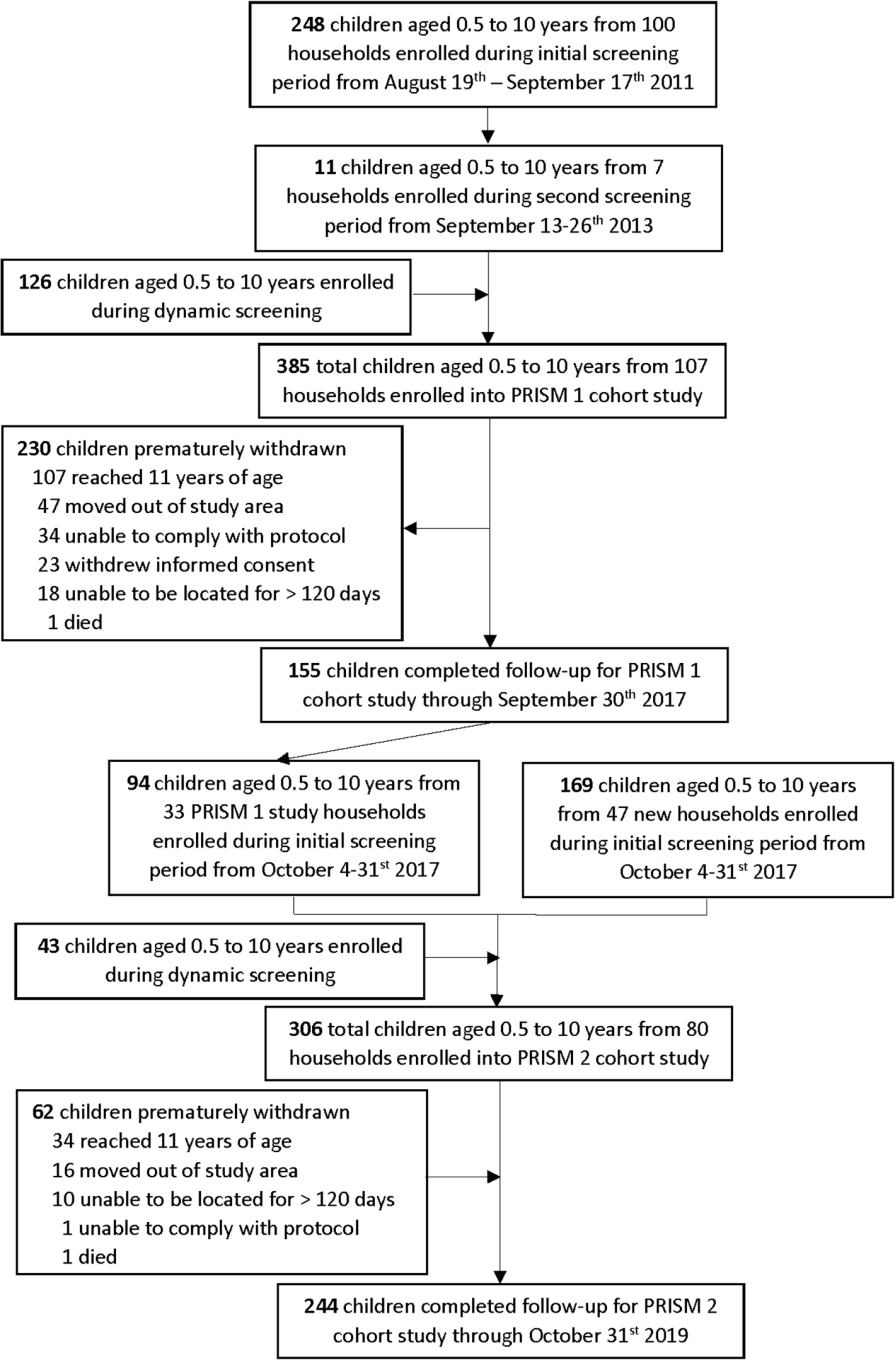
Flow diagram of the cohort studies

Figure 1 summarizes two sequential dynamic cohorts that contributed data to this study. Detailed procedures have been published elsewhere [1]. In brief, children aged 6 months through 10 years of age were enrolled from randomly selected households. The first cohort (PRISM 1) included 107 households and 385 children followed from August 2011 to September 2017. The second cohort (PRISM 2) included 80 households and 306 children followed from October 2017 to October 2019. Thirty-three households and 94 children enrolled in the first cohort were also included in the second. Both cohorts were dynamic such that all newly eligible children were enrolled, and participants that reached 11 years of age were excluded from further follow-up. Parents/guardians were encouraged to bring their child to a dedicated study clinic open every day any time they were ill. Reported seeking of care outside the study clinic for acute illnesses was very rare and comparable across both cohorts (0.2% in PRISM 1 versus 0.5% of annual reported illnesses in PRISM 2). Participating households received free LLINs sufficient to cover all study participants at enrollment and at any time on request at the clinic. Routine visits were conducted in the clinic for all children every 3 months until November 2014, and every month thereafter. The same study clinicians provided care for children throughout both cohorts, drawing from the Uganda Clinical Guidelines (UCG) provided by the Ministry of Health. For both cohorts, detailed medical records were maintained at all visits using the same standardized approach, including history, physical exam, diagnoses, and all medications prescribed.

### Definition of malaria and estimates of parasitemia

At all study clinic visits, children with a history of fever in the previous 24 h or a tympanic temperature ≥ 38.0 °C had an urgent thick blood smear read using light microscopy. If the blood smear was positive, the child was diagnosed with clinical malaria and managed according to national guidelines. At each routine visit, in addition to microscopy, blood was also collected from all cohort participants for the detection of malaria parasites by molecular methods. The molecular methods employed were loop-mediated isothermal amplification (LAMP) in the first cohort and quantitative PCR (qPCR) in the second cohort as previously described [1, 10]. Children with asymptomatic parasitemia (either by microscopy or molecular methods) were not treated with antimalarials, in accordance with local guidelines. At the time of each clinic visit, children were classified as having (1) no parasitemia, (2) sub-microscopic parasitemia, or (3) microscopic parasitemia. If there was no blood smear or molecular test (LAMP or qPCR) performed on the day antibiotics were prescribed, parasite status was classified based on linear interpolation of data from prior and subsequent visits. Briefly, for days in which there were no lab measurements, parasitemia was defined by connecting flanking values of the measured parasite counts with line segments and taking the parasite density on the unmeasured day of interest (i.e., the day antibiotics were prescribed) to be the value along that line. Parasite status for days without microscopic or molecular tests was made using a threshold of interpolated estimates with the classification of days with ≥ 16 parasites/mL as “microscopic,” from ≥ 1 to < 16 parasites/mL as “sub-microscopic,” and < 1 parasite/mL as “no parasitemia.” Additional file 1 contains the full algorithm and flow charts for the parasite density assumptions for the linear interpolation (Additional file 1: Fig. S1) and the classification of parasite status (Additional file 1: Fig. S2).

### Definition of antibiotic treatment and diagnoses

If an antibiotic was prescribed at a clinic visit, this was considered an incident episode of antibiotic treatment. We focused on oral and parental formulations of medications used predominantly for their antibacterial properties; antifungal, antiviral, and antiparasitic medications were excluded, as were non-systemic antibiotics such as topical formulations or drops. Based on standardized diagnostic codes, the primary reasons for prescribing antibiotics were divided into infections by system (e.g., upper respiratory, skin/soft tissue, ear/throat/dental). Antibiotic classes were matched directly to diagnostic codes where possible (e.g., amoxicillin for acute otitis media = ear/throat/dental). If there was no direct match to a category, the best match was determined using associated symptoms (i.e., amoxicillin for ear pain = ear/throat/dental). An “other” category was used if there remained uncertainty as to the reason for the antibiotic or for uncommon occurrences.

### Statistical analysis

Locally weighted scatterplot smoothing (LOWESS) was used to show trends in malaria and antibiotic treatments over time. The primary outcomes of interest were the incidence of malaria and antibiotic treatments per person-year of observation in the cohort studies. The primary exposure of interest was calendar time divided into three periods: pre-IRS (September 2011 through November 2014), years 1–3 of IRS (December 2014 through November 2017), and years 4–5 of IRS (December 2017 through October 2019).

Mixed-effects Poisson regression was used to estimate the risk of a malaria or antibiotic treatment per child per day during each exposure time period [11]. Incidence per person-year was calculated by multiplying incidence per person-day by 365.25. Models were adjusted for age, gender, calendar month fixed effects (to capture seasonal trends), included household- and individual-level random intercepts, and robust standard errors to account for autocorrelated outcomes. An additional analysis fit the same model for antibiotic treatments stratified by six strata representing fever (yes/no) and parasite status (none, sub-microscopic, microscopic). To assist in the interpretation of the magnitude of the trends, measures of association are reported as both incidence rate ratios (IRR) and incidence rate differences (IRD) with 95% confidence intervals (CIs). Statistical analyses were conducted at the 5% significance level and were performed using STATA 14 (College Station, TX).

## Results

### Changes in the incidence of malaria treatments following implementation of IRS

**Fig. 2:**
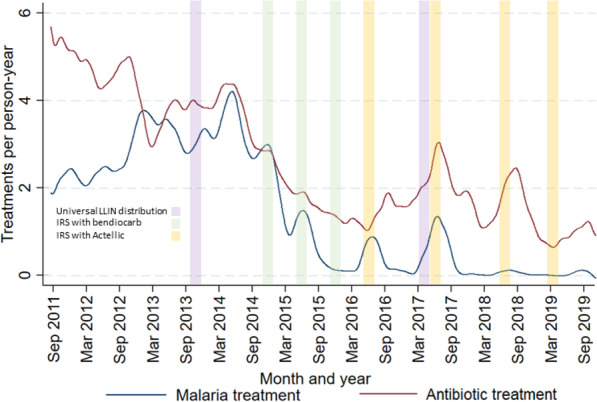
Changes in antibiotic and malaria treatments over time. Daily estimates of incidence generated using LOWESS smoothing

Prior to the implementation of IRS, malaria treatments followed a seasonal pattern with two annual peaks and an adjusted average of 2.68 treatments per person-year (Fig. 2 and Table 1). In the first 3 years following the implementation of IRS, there was a 75% reduction in the incidence of malaria treatments (IRR = 0.25, 95% CI 0.21 to 0.28; *p* < 0.001) with peaks occurring at the time rounds of IRS being delivered. In years 4–5 following the implementation of IRS, treatments for malaria were nearly eliminated with an adjusted incidence of 0.05 per person-year, corresponding to a 98% reduction in the incidence of malaria and 2.63 fewer malaria treatments per child per year compared to pre-IRS (IRD = 2.63, 95% CI 2.36 to 2.90; *p* < 0.001).

**Table 1:** Association between the timing of IRS and incidence of malaria treatments

Time period	Antimalarial treatments	Person-years	Incidence^*^		IRR (95% CI)^†^	*p*-value	IRD (95% CI)^†^	*p*-value
			Crude	Adjusted ^†^				
Pre-IRS	2515	843.0	2.98	2.68	Reference group		Reference group	
Years 1–3 of IRS	455	639.3	0.71	0.66	0.25 (0.21, 0.28)	< 0.001	− 2.02 (− 2.27, − 1.78)	< 0.001
Years 4–5 of IRS	27	489.4	0.06	0.05	0.02 (0.01, 0.03)	< 0.001	− 2.63 (− 2.90, − 2.36)	< 0.001

*Abbreviations*: *IRR* incidence rate ratio (incidence in the comparison group/incidence in the reference group), *IRD* incidence rate difference (incidence in the comparison group − incidence in the reference group), *IRS* indoor residual spraying

^*^Treatments per person-year

^†^Estimates calculated using mixed-effects Poisson regression with robust standard errors. Models included individual- and household-level random intercepts and were adjusted for age, gender, and calendar month fixed-effects

### Changes in the incidence of antibiotic treatments following implementation of IRS

Prior to the implementation of IRS, the average adjusted incidence of antibiotic treatments was 4.14 per person-year (Table 2). In the first 3 years following the implementation of IRS, there was a 53% reduction in the incidence of antibiotic treatments (IRR = 0.47, 95% CI 0.43 to 0.52; *p* < 0.001). In years 4–5 following the implementation of IRS, the adjusted incidence of antibiotic treatments further reduced by 70% from pre-IRS levels (IRR = 0.30, 95% CI 0.27 to 0.34; *p* < 0.001) corresponding to almost 3 fewer antibiotic treatments per person-year (IRD = 2.88, 95% CI 2.54 to 3.21; *p* < 0.001)

**Table 2:** Association between the timing of IRS and incidence of antibiotic treatments

Time period	Antibiotic treatments	Person-years	Incidence^*^		IRR (95% CI)^†^	*p*-value	IRD (95% CI)^†^	*p*-value
			Crude	Adjusted^†^				
Pre-IRS	3534	843.0	4.19	4.14	Reference group		Reference group	
Years 1–3 of IRS	1165	639.3	1.82	1.96	0.47 (0.43, 0.52)	< 0.001	− 2.18 (− 2.48, − 1.88)	< 0.001
Years 4–5 of IRS	636	489.4	1.30	1.26	0.30 (0.27, 0.34)	< 0.001	− 2.88 (− 3.21, − 2.54)	< 0.001

*Abbreviations*: *IRR* incidence rate ratio (incidence in the comparison group/incidence in the reference group), *IRD* incidence rate difference (incidence in the comparison group − incidence in the reference group), *IRS* indoor residual spraying

^*^Treatments per person-year

^†^Estimates calculated using mixed-effects Poisson regression with robust standard errors. Models included individual- and household-level random intercepts and were adjusted for age, gender, and calendar month fixed-effects

**Fig. 3:**
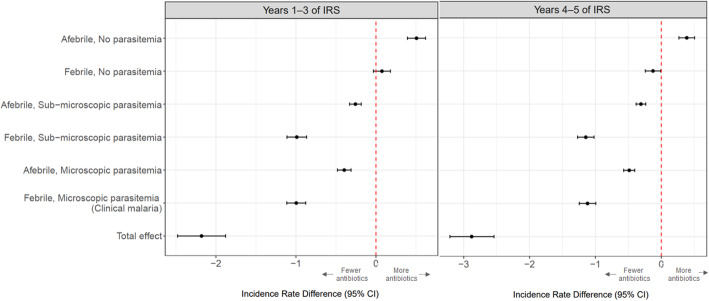
Incidence rate difference in antibiotic treatments compared to pre-IRS levels stratified by fever and parasitemia status. IRS, indoor residual spraying

In the stratified analysis, there was a small but significant increase in years 4–5 in the incidence of antibiotic treatments given when children were afebrile without parasitemia compared to the pre-IRS period (IRD = 0.39; 95% CI 0.27 to 0.51; *p* < 0.0001). All other categories experienced reductions in the incidence of antibiotic treatments 4–5 years after the implementation of IRS. The most marked declines were in the categories of children who were febrile with sub-microscopic parasitemia (IRD = 1.15, 95% CI 1.02 to 1.27, *p* < 0.001) and had clinical malaria (IRD = 1.12, 95% CI 0.99 to 1.25; *p* < 0.001). There were more modest declines among children in the other categories: febrile with no parasitemia (IRD = 0.13, 95% CI 0.24 to 0.01; *p* = 0.036), afebrile with sub-microscopic parasitemia (IRD = 0.31, 95% CI 0.38 to 0.24; *p* < 0.001), and afebrile with microscopic parasitemia (IRD = 0.49, 95% CI 0.57 to 0.40; *p* < 0.001) (Fig. 3).

### Changes in diagnoses and types of antibiotics following the implementation of IRS

**Fig. 4:**
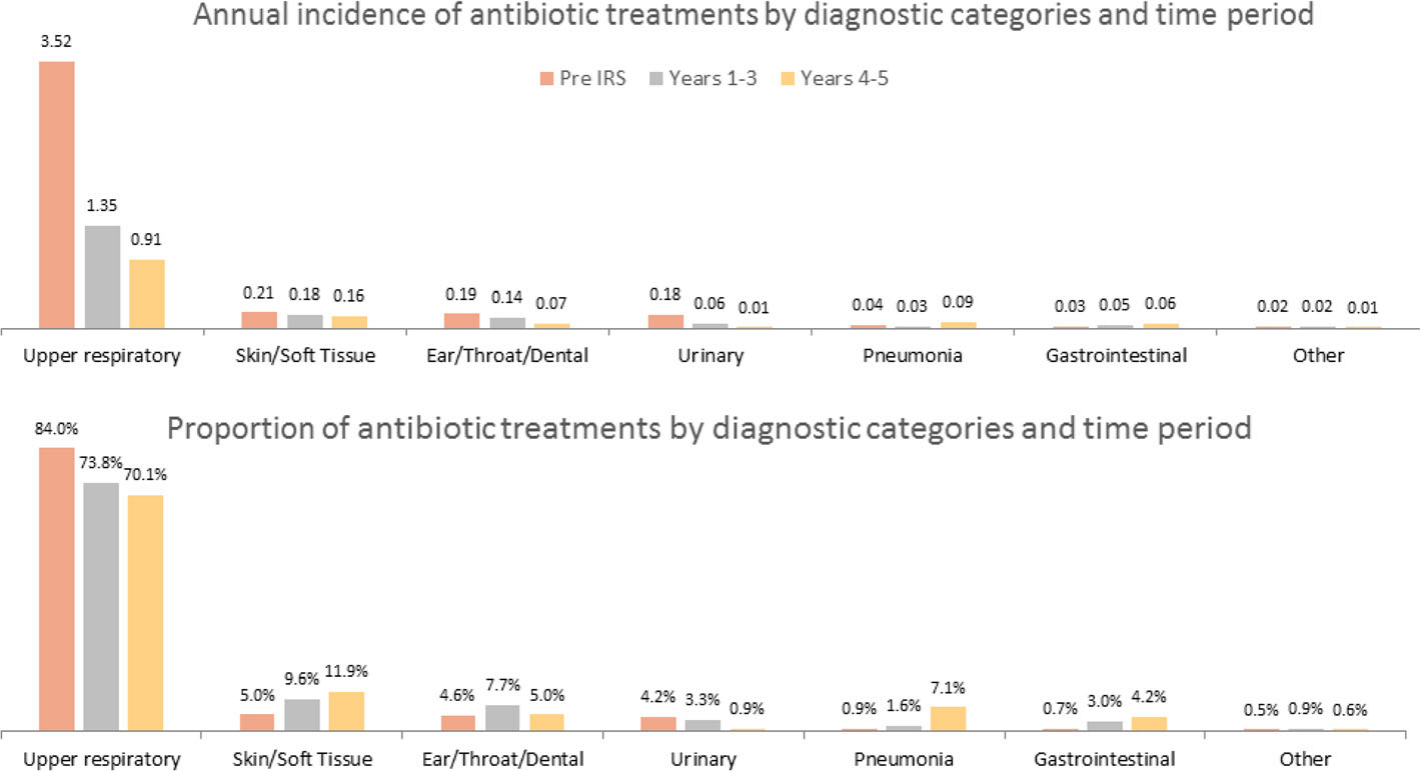
Changes in diagnostic category for antibiotic treatments over time

**Fig. 5:**
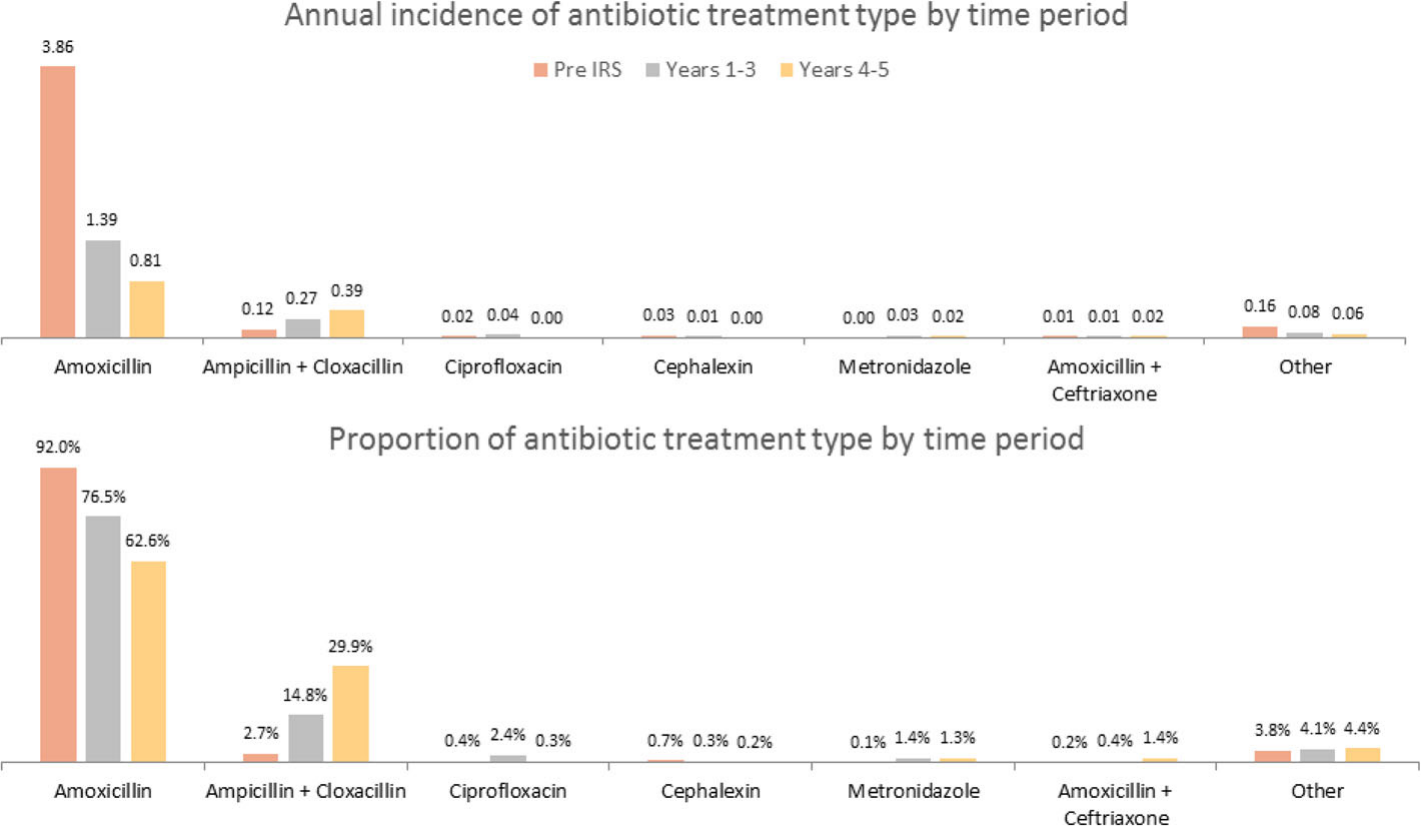
Changes in antibiotic type used for antibiotic treatments over time

Most antibiotic treatments were given for upper respiratory infections (URI), which constituted 84.0% of diagnoses and accounted for 3.52 antibiotic treatments per child per year pre-IRS (Fig. 4). Both the incidence and proportion of antibiotics given for URIs decreased following IRS, comprising 70.1% of visits for which antibiotics were prescribed and an incidence of 0.91 treatments per child per year in years 4–5. The proportion of antibiotics prescribed for skin/soft tissue infections increased (5.0 to 11.9% of all prescriptions by years 4–5), but the incidence remained relatively unchanged (0.21 to 0.16 per person-year). There was a similar pattern for gastrointestinal infections. The incidence and proportion of antibiotic treatments given for urinary tract infections (UTIs) declined over the study period, while treatments for pneumonia increased in both incidence and proportion. Overall, amoxicillin was the most common antibiotic prescribed, accounting for 92.0% of antibiotic prescriptions and 3.86 per child per year pre-IRS (Fig. 5). Mirroring the pattern for URIs, amoxicillin prescriptions also decreased in both incidence and proportion following IRS, comprising 62.6% of antibiotic prescriptions and 0.81 amoxicillin treatments per child per year in years 4–5. Ampicillin plus cloxacillin increased in both relative proportion (from 2.7 to 29.9%) and incidence (from 0.12 to 0.39 treatments per child per year) by years 4–5. The use of other antibiotic types was uncommon.

## Discussion

In this highly malaria-endemic region of Uganda, successful malaria control with LLINs plus IRS was followed by almost complete elimination of symptomatic malaria and a substantial reduction in the incidence of antibiotic treatments among children 0.5 to 10 years of age. Indeed, in the 4–5 years following the implementation of IRS, children received nearly three fewer antibiotic treatments per year, a 70% reduction compared to the pre-IRS period. These results provide further evidence that in highly endemic settings, successful malaria control efforts can have substantial health benefits for children beyond those directly attributable to reducing the burden of symptomatic malaria.

In addition to the important health benefits to the children and the socioeconomic benefits to families, the magnitude of reduced antibiotic treatments demonstrated in this study has important implications for health systems in malaria-endemic settings. Countries like Uganda struggle with training and retaining staff to ensure quality care and meet patient needs [12]. Intensive malaria control with LLINs and IRS in this region was associated with 2.6 fewer treatments for malaria and nearly 2.88 fewer antibiotic treatments per child per year. At a health system level, this could result in a substantial reduction in patient volume, staffing needs, and costs associated with care. Furthermore, the high number of antibiotic treatments averted, mostly amoxicillin, could reduce selection pressure and help slow the spread of antimicrobial resistance which is a major public health threat in Uganda and elsewhere [13, 14].

There are multiple plausible mechanisms explaining the association between effective vector control leading to reduced malaria burden and an accompanying reduction in antibiotic use among children. As malaria burden decreased precipitously, the incidence of antibiotic treatments for all categories of children with parasitemia decreased compared to the period before IRS was implemented. This decrease was most marked among febrile children, such as those diagnosed with clinical malaria or were febrile with sub-microscopic parasitemia. There was a more modest decrease for children with parasitemia who were afebrile. This pattern of greater decreases in antibiotics prescribed for febrile versus afebrile children was observed across all categories of parasitemia. In general, patients diagnosed with malaria may also be prescribed antibiotics because of concern for other co-occurring illnesses. Indeed, in this study, 40% of children treated for malaria were also given antibiotics prior to the implementation of IRS, which decreased to 20% after the implementation of IRS. Thus, reducing the incidence of malaria may reduce susceptibility to concomitant illnesses and limit opportunities to prescribe concomitant antibiotics [5]. Sub-microscopic parasitemia may induce symptoms, including fever, triggering clinic visits that may result in an antibiotic prescription, particularly if the parasitemia is below the level of detection by standard laboratory tests and the clinician is unaware that the patient is infected with malaria. Other contributing factors that play into a clinician’s decision to prescribe an antibiotic may be independent of clinical symptoms. In cases where a clinician is unsure of the utility of antibiotics, clinicians often face an expectation to provide some form of intervention or treatment as a conservative measure or to meet patient demands [15]. Perceptions of risk for the progression to severe disease and trust within the clinician-patient relationship also play important roles [16].

As malaria burden declined in the population following IRS, one might have expected an increase in the incidence of antibiotic treatments among the febrile children who tested negative for malaria by microscopy as has been found in other low transmission settings in Uganda [6]. However, we found that the incidence of antibiotic treatment decreased slightly for febrile children without parasitemia and increased for afebrile children without parasitemia following the implementation of IRS. It is possible that clinicians felt pressured to provide antibiotics for afebrile children, even for those with lower severity illnesses such as viral URIs for which antibiotics are typically not recommended, since they had nothing else to offer. On the other hand, febrile children received an urgent blood smear according to the study protocol. This diagnostic test may have fulfilled the expectation to provide some sort of intervention and allowed the clinician to forgo an antibiotic prescription. These results contrast, however, with multiple studies showing antibiotics are typically prescribed more frequently with negative malaria tests [6, 17–19]. In this cohort study, repeated contact may have allowed clinicians to develop stronger relationships with families, making it easier to avoid antibiotics overall. It is also possible that the children may have become healthier over time solely due to the rigorous free health care provided as a part of the research study or that exposure to other bacterial or viral illnesses in the community, which we did not measure, may have also been declining. Future research is needed to explore antibiotic prescription practices relative to parasitemia status and fevers in more typical clinic settings.

Another potential mechanism underlying the reduced antibiotic treatments found here may be reduced susceptibility to co-infection with acute bacterial, viral, or other parasitic infections as a result of decreased parasitemia [20]. An association between malaria and serious bacterial infections has been documented among severely ill children in hospital settings [21]. While less well studied, malaria may also increase the risk of less severe infections seen in outpatient settings through indirect effects on the immune system [20], anemia [3], birth weight [22, 23], and nutrition/growth [24]. Indeed, malaria infections at any density have been associated with serious health outcomes [25]. Furthermore, malaria control may improve overall child health by mitigating the socioeconomic costs incurred by the household due to malaria-related illness [26]. Finally, malaria control targeting mosquitoes may decrease the risk of other vector-borne illnesses, such as arboviruses or other parasitic infections, though studies on these types of pathogens are limited. This could be one potential explanation for the decrease in the incidence of antibiotics among children who were febrile and non-parasitemic in our study, such that intensive malaria control (i.e., IRS) may have reduced the risk of other vector-borne illnesses and thus avoiding another opportunity to prescribe antibiotics for a febrile non-malarial illness.

This study had several limitations inherent with observational studies making “before and after” comparisons. First, we lacked a contemporary control group which did not receive intensive malaria control, thus limiting our ability to make causal inferences. Second, there are limitations with external validity given that our study participants were enrolled in a research study and provided more rigorous care than is typically available in resource-limited settings. However, the extended follow-up and the consistency inherent in clinical management by only a few providers using highly standardized procedures are benefits of the cohort study design. Third, our analyses did not adjust for secular trends in malaria and antibiotic treatments, as there is a complex relationship between these and seasonal variations making accurate modeling of these trends challenging. Though we are unaware of other significant changes in the district, it is possible that other health programs or other changes in the study area could explain the decrease in antibiotic use over time. Finally, the stratified analysis partially relied on estimates of daily parasite status interpolated from prior and subsequent lab results, potentially introducing bias.

## Conclusion

Intensive malaria control in this previously high transmission setting was associated with a significant decline in the use of antibiotics, as well as malaria treatments, in a closely followed cohort of children. We found that investments in malaria control may have broader health benefits for children beyond malaria. Future studies should investigate the association between malaria control and antibiotic prescribing practices in other settings to further explore the impact on health systems and to inform strategies to mitigate the overuse of antibiotics and the threat of antimicrobial resistance in malaria-endemic settings.

## Supplementary Information

**Additional file 1: **Algorithm for classification of parasitemia status. **Figure S1.** Parasite density assumptions for linear interpolation. **Figure S2.** Classification of parasite status.

## Data Availability

The datasets used and/or analyzed during the current study are available from the corresponding author on reasonable request.

## Abbreviations

CI: Confidence interval
LAMP: Loop-mediated isothermal amplification
LLIN: Long-lasting insecticide-treated bednet
LOWESS: Locally weighted scatterplot smoothing
IRD: Incidence rate difference
IRR: Incidence rate ratio
IRS: Indoor residual spraying
qPCR: Quantitative polymerase chain reaction
UCG: Uganda Clinical Guidelines
URI: Upper respiratory infection
UTI: Urinary tract infection
