# Supplementary material for: Marked reduction in antibiotic usage following intensive malaria control in a cohort of Ugandan children
Source: BMC Med. 2021 Nov 30;19:294. doi: 10.1186/s12916-021-02167-2 (PMC8630830; doi:10.1186/s12916-021-02167-2)

Additional file 1

Algorithm for classification of parasitemia status

Children were classified as having 1) no parasitemia, 2) sub-microscopic parasitemia or 3) microscopic parasitemia for each day, either from direct lab results or estimated via an interpolation method, according to the following procedure (**Figures S1 and S2**):

1. If a child had a positive blood smear, they were classified as having "microscopic parasitemia" and the parasite density on that day recorded as the parasite density read directly from the blood smear. **STOP**
2. If there was a negative blood smear on the day of an antibiotic prescription…
    - **a.** …and the LAMP result was positive, then they were classified as having "sub-microscopic parasitemia" and the parasite density was assumed to be 10 parasites/mL. **STOP**
    - **b.** …. and a qPCR value >=1 parasite/mL, they were classified as having "sub-microscopic parasitemia" and the parasite density was assumed to be 10 parasites/mL.
    - **c.** …and the LAMP/qPCR was also negative, then they were classified as having "no parasitemia" and the parasite density was taken to be 0 parasites/mL. **STOP**
3. The parasite density for children who were treated for malaria was assigned to 0 for the next 14 days.
4. For days in which there were no lab measurements, a linear interpolation method was applied to estimate the parasitemia status. This method connects values between measurements with line segments and takes the parasite density on the unmeasured day to be the value along that line. A lower limit of 16 parasites/mL was assumed to be the cutoff for a positive blood smear or "microscopic parasitemia".
5. On a day with a negative or missing blood smear and…
    - **a.** …either 1) the qPCR or 2) the parasite density estimated via ipolation was >=16 parasites/mL, the child was designated to have "microscopic parasitemia". **STOP**
    - **b.** …one of 1) a positive LAMP result, 2) a qPCR-measured parasite density >=1 and <16 or 3) an ipolated parasite density >=1 and <16, the child was designated to have "sub-microscopic parasitemia". **STOP**
    - **c.** …and either 1) the qPCR or 2) ipolated parasite density was <1, the child was designated to have "no parasitemia". **STOP**

In this fashion, the parasitemia status for each child was classified for every day on which they received an antibiotic prescription.

Figure S1:

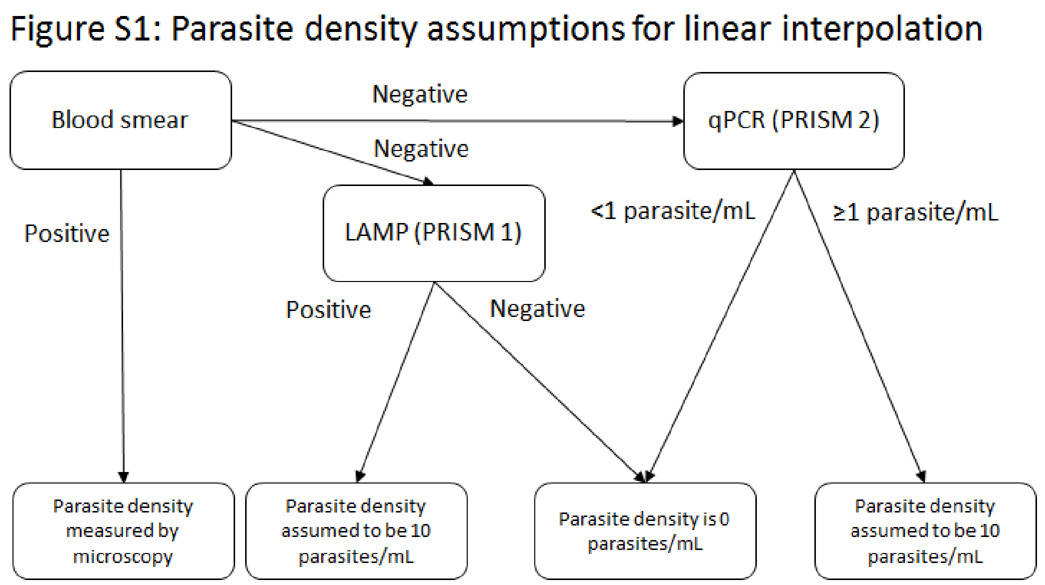

Figure S2:

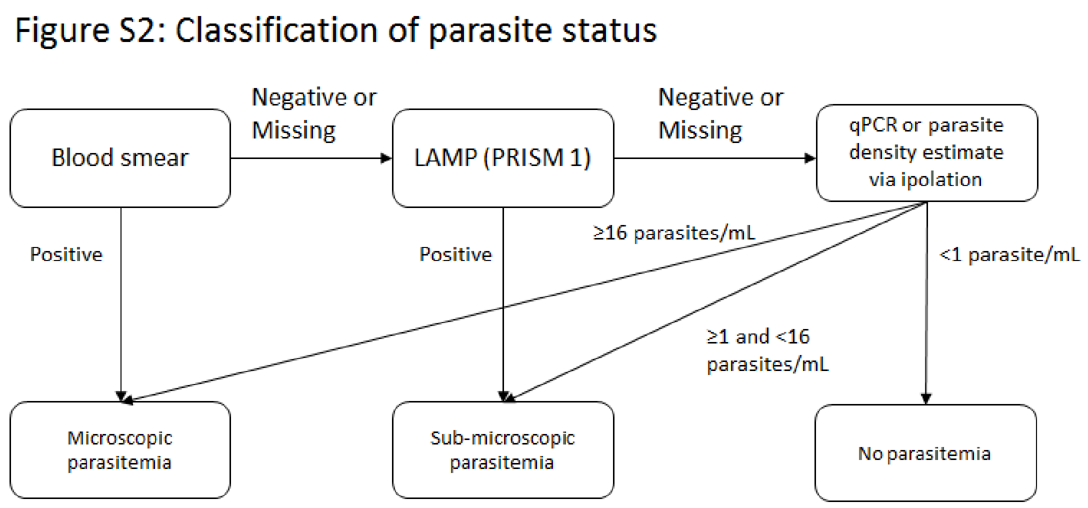

Supplement: Supplementary file 1 — Additional file 1: Algorithm for classification of parasitemia status. Figure S1. Parasite density assumptions for linear interpolation. Figure S2. Classification of parasite status. [file 12916_2021_2167_MOESM1_ESM.pdf]
